# Endangered indigenous knowledge: Assessing locally adopted tree conservation practices in Aleta Wondo district, Sidama regional state, Ethiopia

**DOI:** 10.1016/j.heliyon.2023.e15401

**Published:** 2023-04-14

**Authors:** Tirfu Kakiso

**Affiliations:** Lecturer at Hawassa University, Geography and Environmental Studies, Ethiopia

**Keywords:** Indigenous knowledge, Endangered, Tree conservation, Agro-forestry

## Abstract

Environmental quality cannot be maintained unless locally adopted indigenous knowledge of environmental conservation practices is duly considered. This study is, therefore, aimed to assess major indigenous practices and threats of indigenous tree conservation practices of Sidama people particularly focusing on Aleta Wondo district. It was also intended to investigating the factors influencing the continuity of practices in the vicinity. In order to collect the data, the local elders and rural development work agents were consulted as primary data sources. Both published and unpublished materials such as documents, journals, articles, books and official reports were accessed as secondary data sources. The research was conducted primarily by employing qualitative methods both for data collection and analysis. Based on the collected data, the major indigenous tree conservation practices in the study area include the places like kakkalo site, grave yard, sacred site, Gudumale and agro-forestry in the farm garden. It has also been identified that indigenous practices of conserving larger trees is declining due to the influences of religion, increasing living cost, education system and population growth. Moreover, no significant intervention was found exercised to tackle the problem. As a result, it is recommended that such locally adopted conservation practices should be addressed effectively in formulation and implementation of nationwide policies and strategies.

## 1 Introduction

The community in every pocket of the world has their own rich culture of caring for their surrounding environment [1]. In support of this [2,3], stated that local people in different parts of the world have adapted their lives to the circumstances of the physical world around them. These cultures, most often, termed as indigenous knowledge (hereafter IK) have contributed a lot to the maintenance of the sustained environment for most parts of human history [[4], [5], [6]]. In this regard [7,8], confirmed that IK has helped the local people to manage their survival in different circumstances. The IK has been developed and adapted to the gradually changing environment and therefore, it is the social capital of the poor as well as an asset for investing in the struggle for survival. Mainly due to their deep-rooted knowledge of their surroundings and their relationship with the environment, it helped the local community to find ways to resist and adapt to environmental changes, whenever it happened.

These cultures, most often, termed as indigenous knowledge (hereafter IK) have contributed a lot for the maintenance of the sustained environment for the most parts of the human history [4]. Indigenous knowledge is, therefore, a valuable national resource to enhance sustainability of development and land use systems closely intertwined within their culture and well adapted to their ecosystem [9]. Considering this fact, has stated that managing environmental problems (particularly climate change) requires holistic understanding of the current conditions including the cultural traditions of the community.

In addition, it has been confirmed that the early societies, being highly dependent on their physical surroundings, developed their own way of managing the beauties of the environment: all forms of natural resources like forest, soil, water, climate and so on [10]. Owing to this reason, the problems with the environment that our generation is yelling about have never been serious issues, at least not until the beginning of the second half of the 20th century [11]. This might be because our ancestral communities developed different mechanisms for the maintenance of the quality of natural resources with consideration of intergenerational equity [12]. Has ascertained that long before the development of modern forest science and ‘scientific’ forest management approaches in Europe in the early nineteenth century, local and indigenous communities throughout the world managed forests and associated landscapes in countless ways that sustained their livelihoods and cultures, without jeopardizing the capacity of these ecosystems to provide goods and services for future generations.

However, nowadays, environmental sustainability has not been given due attention. It might be because of the fact that the unprecedented growth of the human population [13] and its associated consumption is accompanied by advanced technology and competition for domination of the economic world [14]. This can be, in short, expressed as attempting to bring economic progress at any negative cost to the environment. Most of the inhabitants of the current world have been striving to strengthen their power either to maintain their household's food security [15,16] or produce more and more income with advanced technology so as to maintain economic superiority over others. Such striving for economic production has proceeded by being ignorant not only to the sustainability of environmental resources but also to the needs and demands of the future generation [15]. Consequently, diversified and multifaceted environmental problems such as climate change, land degradation, water pollution, loss of biological diversity and deforestation have been commonly experienced by the global community in varying magnitudes [14].

The problems are becoming the worst because of the deterioration of the local ecological knowledge maintained for centuries [17]. Local ecological knowledge, in this paper, alternatively used for IK, expresses the local knowledge and practices developed for the management of the environment [2,6,18] and maintained for several years by the local people. Local ecological knowledge has been very helpful for the continuity of the quality of ecological services at the same time addressing the communities' livelihood interest [12,17,19,20]. In today's remnant of the forest over the world, the IK of the people living around the forested region has the grave footprints [12,21]. However, currently due to the declining acceptance and practice of the IK not only the forest is facing declining effect [22] but also the totality of ecosystem has been facing the challenges of extinction [12,23].

In Ethiopia, what have sustained the quality of the environment for long is not the efforts of the government or the overwhelming scientific approaches of environmental management but locally developed and adapted practices and knowledge in different parts of the country [19,20,[24], [25], [26], [27]]. This can be best exemplified by the Agro forestry system of Gedeo, Sidama and Bench Maji; terracing practice of Konso; mulching techniques of Derashe; crop rotation and cropping in different districts of the country. In most cases, integrated indigenous environmental management activities are undertaken; i.e more than one indigenous practices are put in place when managing the surrounding natural resources by the people [26].

The Sidama Community, currently found in the Sidama region with its regional capital, Hawassa, is situated in the southern part of Ethiopia. Being located at 273 km away from the Ethiopian capital, Addis Ababa, the region is well known by numerous indigenous practices, including Fichee Cambalaala, a UNESCO inscribed intangible heritage. Like its brother communities in the region and the surroundings, Sidama has developed normative and well-thought practices in relation to environmental management. Different local-based indigenous practices, but not completely confined to them, are implemented by the community, making tremendous contribution to the quality of the environment [20,24,27,28]. Among different indigenous practices, indigenous tree conservation techniques are commonly practiced by the local communities of Aleta Wondo district. However, it became vivid that currently both the indigenous practices and tree coverage have been showing a declining trend. Even though the fact on the ground shows that such indigenous practices are decline and at the brinks of complete completely lost, no researchers are conducting to on the issues in the district under consideration. The researches conducting in other parts of the country also do not explicitly implicate the major driving factors of the declining trend of indigenous practices. It is therefore, this rationale that necessitates conducting this study. Thus, this study tries to address the indigenous practices of tree conservation of the Sidama people and aimed to investigate the factors contributing to the declining trend by taking Aleta Wondo district as a study area. It can be understandable that this study more relied on described the existing indigenous practices in the study area and pointed out major threats for the sustainability of the practices. It is believed that the study would have benefited much if statistical approaches could have been employed. However, this study was conducted by employing more of qualitative methods and therefore, the level of significance of factors was not treated.

### 1.1 Objectives of the study

This study is generally intended to assess the fate of indigenous practices of tree conservation among the Sidama community in Aleta Wondo district. Specifically it is targeted to achieve the following objectives.

➢ To describe indigenous tree conservation practices of the community in the site of study
➢ To identify major factors negatively affecting indigenous practices of the tree conservation in the area under consideration

## 2 Methods and materials

### 2.1 Study Area description

**Fig. 1:**
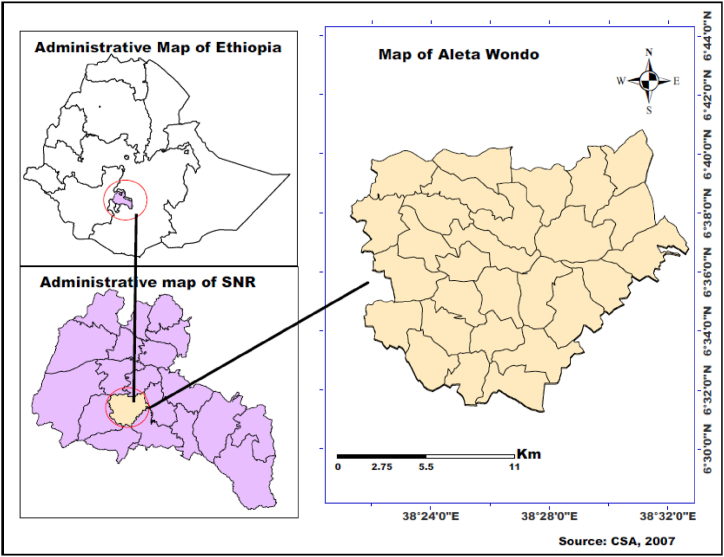
Map of study area.

Aleta Wondo *district is* one of 36 rural administration structures in Sidama region, which situated in central geographic position a little inclining towards south. The district is located at about 65 km in south of Hawassa city, regional capital and 338 km from Addis Ababa, the capital of Ethiopia. With respect to its absolute position, it is situated between 6°31' - 6°41′N latitude and 38°21' - 38°32′ E longitude (see Fig. 1). The current population of the district is 275, 842. The district is further sub divided into 27 smaller administrative structure called *kebeles* and its administration centre is Aleta Wondo town. Geographically, Aleta wondo district is bordered by Dale in north, Bursa in south, Hula in the south east and Aleta chuko in the west and Otilcho in the south west direction. Total land area of the district is 27, 873ha, among which 64% is covered with permanent crop, 25% annual crops, 8% covered by natural, cooperatives and private forest, 2% covered by grazing land and remaining 1% of land is covered by others. Furthermore, district is known for production agro forestry practice which make greener, tough declining trend can be traced. As far as agro-climatic condition is concerned, 37% of landmass of district constitutes highland/*dega* agro-climatic condition while the remaining 63% is categorized under midland/*woyena dega* agro-climatic zone. Annual temperature of the woreda varies between 15.1c to 22.5°c while the annual rain fall ranges between 1200 mm and 1600 mm.

### 2.2 Research methodology

The research design employed to address the above stated objectives is more of descriptive, following a qualitative approach. For this study, local elders, rural development agents and the physical environment are used as a source of data. As part of data collection methods, interviews and focus group discussion were conducted. Accordingly, about 19 elders aged between 51 and 67 who are supposed to have rich experiences in the traditional practices of the communities under the area of focus have participated in the interview. In addition, key informant interview has also been conducted with the district level rural environment protection officers so as to substantiate data collected from local elders. In both case, the researcher employed semi-structured interview in which some questions were listed, however, depending on its necessity, some addition questions were posed for further clarity. Moreover, two in depth focus group discussions, each composed of 6 diversified individuals including both sex, have been employed where the researcher raised point of discussion guided it. Furthermore, the observation of the existing environment, particularly trees in target area was used as the other method of data collection. Finally, since data collected were qualitative in its type, qualitative method of data analysis was carried out where narrative description mostly employed though graph is also used in few cases.

## 3 Results and discussion

This section of the research involves analysis and discussion of the data gathered employing mainly qualitative techniques. The data shows that Sidama communities have a rich culture of caring for their surrounding environment in general and trees in particular. As discussed in the upcoming sections, most of the cultural beliefs and practices of the community are highly in touch with the protection of the environment. Among many, some of the practices were identified to be the major ones by local people engaged in interview and focus group discussion are described here below.

### 3.1 Indigenous practices of the tree conservation

#### 3.1.1 Kakkalo site

In most cases, larger trees are not let to be cut for any purpose because it is believed that they are the only place under the shed of which *Kakkalo* (literally translated as sacrifice) is offered to their *Magano* (God) and to their ancestral fathers. With this respect, it has been mentioned, by Key informants and focus group discussion with the community selected representatives, that in Aleta wondo district people of any clan in the site of concern slay the oxen or lamb of sacrifice to their protector and saver *Magan* (God) under the shed of larger trees most specifically the tree locally called *Dagucho (Afrocarpus falcatus).* Because, those larger trees are considered by the local people to be ritual place, they are not allowed to cut for any purpose.

A study conducted by Ref. [1] has plainly indicated that Songo sacred trees are often seen as ritualistic trees which are maintained and protected by prohibition systems or taboos. The informants of the study, in Aleta wondo, have mentioned that a person who violates the rule of community by cutting off those sacred trees for his/her own intention is subjected to punishment based on the norm of the community in the locality. According to the interview conducted with the wereda rural development and environmental protection officer, and development agent of the kebele, larger trees are mostly found currently in areas where “traditional” practices like Kakkalos are performed by the local community. Thus, it can possibly be implicated that Kakkolo, as the indigenous practices of people in Aleta wondo and generally in Sidama, has contributed to the sustainability of larger trees and the environment in a broader sense. This finding goes hand in hand with [29] finding which states that Indigenous knowledge of communities in Gamo areas has played a significant role in maintaining biodiversity.

As a result, it is uncommon to cut larger trees from these types of sites, according to community belief. However, as key informants in the study, the interviewees did not want to hide the fact that the trend of these practices is declining and that they are now rare. Thus, everyone is claiming the trees found on his plot of land belong to him, and no one can prohibit them from using his own property bylaw.

#### 3.1.2 Grave yards

The interviewees and FGD discussants commonly agreed that the Sidama people in the district under consideration are known to conserve any trees, regardless of their kind or species, grown in and around burial sites (grave yards). The concern to conserve the trees around graveyards is high as the intimacy or blood relationship of the deceased person becomes closer [20]. Has associated this with the community's belief in ancestral spirits. He further assured, based on the interview conducted with the officers, that individuals in the community can maintain a specially designated graveyard where the dead would be buried and regard the graveyard as sacred. When a renowned ancestor dies, local people in the Aleta Wondo district believe that the spirit of the ancestor lives on with the progeny. They venerate the ancestral graveyard by offering Xorsho (pouring honey mixed with curd milk on the tomb of the deceased), mead, and other sacrificial items [25]. Assessing the Indigenous knowledge of Oromo, the people who is very tight with Sidama in most cultural practices, has confirmed the intention of protecting trees in burial site as:

“*burial sites are historical and memorial places for the clan of the deceased to remember the individual of their clan. These places are also part of clan land. The community believes that for the deceased, this land was their resting place and their Ayyana resided around. The value and assumptions the community attaches to this land serve to protect and conserve this land and the vegetation it supports. It is believed that an individual possesses Ayyaana (spirit) while alive, and that when the person dies, this Ayyana (spirit) remains on his grave*".

As to the response of participants in FGD, the one who uses trees grown around the tomb of his deceased relatives is considered the one who is ruthless and even immoral. In this case, there is no set punishment rule, but there is a social morality attached to it, and any member of the community who goes against the unwritten norm of the society is considered abnormal from the cultural perspective of the people.

#### 3.1.3 Sacred place

In Sidama, it is not uncommon to see larger trees around traditional religious intuitions whose belief is based on the patriarchal ancestral fathers *Abbo* (the earlier father of the *Holloo-Garbicho* tribe), *Tumano* (the earlier father of the *Yemerero* and *Fakisa* tribes), and *Akaako* (the earlier father of the *Alata* tribe). In those ritual places, where people not only believe and worship their gods for their eternal life after death but also for bringing peace and harmony among the community, larger trees are used as houses for meetings. Hence, cutting trees from such ritual places is considered to be against *Halaale* (literally translated as “truth,” but it is beyond that). The researcher himself got the chance to physically observe *Abbo* religious practice in the district, specifically in *Wotto kebele* (Kebele is the lowest administrative structure in Ethiopia), where there was a large congestion of trees and elders gathering to reconcile the disagreement between individuals. This study is found to be congruent with [20] finding that confirmed *Abbo* religious practice in *Wonsho* has a great contribution to the conservation of biodiversity in the region of his focus [11]. On his part, confirmed that a sacred area is forested and is associated with deities' (mostly ancestral spirits') rituals and taboos [28,30]. similarly, asserted that in traditional protected areas not only trees but also wildlife are safeguarded because of their sacredness or spiritual significance to custodian communities. Moreover [11], revealed that the traditional customary rules prohibit the felling of trees and the killing of animals in the sacred sites. Such customary laws and rules of the local community have contributed to the conservation of trees in Aleta Wondo.

#### 3.1.4 Gudumale

The place where frequent meetings are held and holidays like *Fichee-Cambalaala*, a New Year celebration based on a lunar calendar, take place, is also the place where collections of larger trees are found. In addition to such ceremonial activities, the same site is used to hold meetings. In this regard, the elders of Sidama have their own traditional parliamentary or decision-making process, which is carried out not in a built hall but in the place called *Gudumale*, under the shady shelter of huge and numerous larger trees, mostly under Dagucho (Afrocarpus *falcatus*) and *Hooncho* (*Juniperus procera*), intentionally left for such public practices. Because of their public values, any tree growing in the place called *Gudumale* is not subjected to cut for any purpose since cutting trees is considered a violation of the rule of *Haalale*. The place is called communal, and the trees are too. So that, every member of the community, irrespective of his age and clan, cares about and for the trees in that holly place. The writer of this research has observed two *Gudumales* located closer to Aleta Wondo town with larger *Dagucho* (*Afrocarpus falcatus*) trees. Based on the participants' and the researcher's physical observations, it is clear that there is a downward trend in the conservation of trees in such communal places. This has been similarly reported by Ref. [31] that *Gudumal*e has a significant role in conserving varieties of plant species.

Moreover, most interviewed elders replied that it is common practice among the Sidama community not to uproot seedling in the farmyard when tilling or hoeing and harvesting. In this case, seedling like Dagucho*(Afrocarpus falcatus)*, *Waadicho* (*Cordia Africana)*, *Masincho (Croton macrostachyus)* and *Gidincho (Ehretia cymosa)* are given great care for different environmental, social and medicinal purposes. The intention of caring for those plants is that they have larger leaves and can shed crop from the impact of sunlight and gradually decomposition of the leaf is also considered to be a huge source of organic matter next to dung. Farmers in early days, until 1990s, are not familiar with chemical fertilizers and mostly rely on organic manure and biological treatments. The researcher himself observed plants of different species in the farmyard of the local communities which can best exemplify the agro-forestry practice with bio-diversity conservation. The participants in the study agreed that this home garden agroforestry is still common in the study area but is showing a declining trend.

### 3.2 Threats for indigenous tree conservation practices

The reply from the key informants showed that such beautiful cultural practices are declining from time to time. As to the agreement of FGD discussants, nowadays, there is frustration that the continuity of such environmentally friendly behavior is at risk. The interviewees have also indicated that the aforementioned practices are currently rarely practiced. If this continues at the same pace, they will be completely lost in the future, which will exacerbate future environmental problems. Hence, it can be said that indigenous knowledge of conserving the environment generally and especially the aforementioned practices are endangered currently. As all respondents commonly agreed, a decline in indigenous cultural practices is happening because the youngsters have placed less emphasis on their families’ norms and cultures. Among the major factors responsible for the deterioration of these indigenous practices of caring for the plants, the following are found to be the most important ones.

One of the factors for the decline in indigenous practice, as participants indicated, is conversion of religion. As to the respondents of reply key informant interview and FDG, most cultural belief and practice are at the verge of extinction due to the influence of religious conversion from the traditional *Halaale* system to Christianity predominantly Protestantism and to lesser degree Catholic and Ethiopian Orthodox Christianity [32]. has non-ambivalently stated that greater than 3/4th of the population in the Sidama vicinity found to follow protestant and Catholic Christianity, about 10% follow Ethiopia Orthodox Christianity, the nearly the same proportion of Muslim were reported while only less than 5% in vicinity practices traditional religion. [33], conducting study on “state –society relationship and traditional governance in Ethiopia” has attested that among the challenges that Sidama traditional political institutions facing is that intrusion of different denominations of Christianity, and the declining interest of the youth in terms of adherence to the traditional values and norms. Moreover [34], confirmed most traditional practices of the Sidama people have been declining due to the introduction of the new religion through conversion of traditional religion followers to Protestantism dominantly. In line with this, most of the interview participants assured that Protestant religion condemns most traditional practices of indigenous society (Sidama) considering it as backward tradition which goes against the principle of biblical philosophy. People in the area were wrongly oriented that any traditional act whatever its kind is and how much it positively contributes, protestant religion teaches that the only one to be praised is God, Jesus who does not like any traditional culture. In this case, providing sacrifice to the ancestral and deceased father is now considered to be sign of evil ghost. So that, the values of trees that were used to provide the offering under the shed of it is now completely lost. Hence, larger trees now a day given no care and who he is thought to be the owner of it can do whatever he likes.

Most participants of this research claimed that currently because of the increasing living cost larger trees are sold for survival. People in the district have maintained their own lifestyle and ensured their food security for long time with their substance and to some extent commercial based agriculture. However, currently owing to the fact that climate change, failed political commitment and above all, fast growing population, the production capacity of farmers and productivity of land has already been declined. As result, living cost for dwellers is increasing and becoming among the challenges for the sustenance of the household income. As a response to this, now day, it becomes common practice that local community in the area of focus have been using those larger and most respected trees for family consumption. For example one of the discussants using his own experience elaborated “at every September I used to sale *Dagucho* from my farm yard for lumbering because I need money badly to fulfill my children's education material requirement”. In addition, the monetary value of tree increased: single large tree may cost/earn more than 3000 Eth birr (which is estimated to be nearly $112 U S). So that monetary values of larger trees, now a day, is considered to be by far greater than its moral values. This in combination with declining production of agricultural land and livestock farming has put serious endanger on the existing of trees that have both environmental ad aesthetic values.

It is also clearly identified that youngsters basically who attended modern education give no or little attention to such traditional practices wrongly considering it backward and uncivilized. Most of the interviewees of this research have confirmed that none of the children who got a chance to attend modern education and employed in government sectors are giving emphasis on growing trees and they highly incline to urban way of life”. As to FGD discussion, the same holds true to most community in the area of consideration. Even the writer himself has recognized the same gap from his own friends from informal discussion about the issues. Equally challenging to most indigenous knowledge to sustained is that the focus given in formal education system. Education system of the country highly replied on the western (scientific) knowledge. Little or no attention is given to teaching the children the fact they grown up through the knowledge and practice they are born in. As a result strong bondage with community knowledge has been losing more inclining to the curriculum probably copied from foreign countries given less consideration to the existing fact in a locality.

**Fig. 2:**
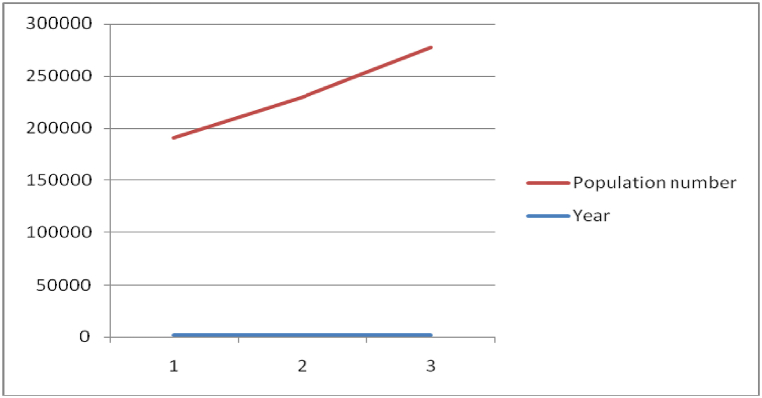
Population growth trend in the study Area.

Moreover, the total population of the district is increasing over time which in turn influences the chance of practicing indigenous tree conservations. As indicated in (Fig. 2), total population of the district was 188,976, 227,910 and 275,568 in 2007, 2014 and 2021 respectively. This rapid growth of population has resulted not only on the decline of farm holding size but also productivity of land due to frequent cultivation of the small plot of land to support their substance way of life. For instance, the density of population in 2007 was 819 persons per square kilometer [32] and projected to reach nearly 1195 persons per square kilometer [35]. Since the production and economic benefits from their plot of agricultural land is minimal, caring about the futurity of the environment may be considered as luxury. The increasing population, therefore, incur greater demand for the farming land and leads to deterioration of tree covered both from farmland and the surrounding sacred groves in the region. Moreover, due to the increasing demand to food and shelter, as a means of survival, not only trees but also every asset including land holding is subject to sale.

In this regards [36], revealed the impact of population growth is a problem in environmental recovery though not only critical. A study done by Ref. [37] plainly stated that due to high population growth, agricultural practices have expanded into upland and marginal areas, and clearing of indigenous trees has become prevalent. Thus, the current population puts the existing tree conservation practices under pressure and gradually causing the environment at risk of degradation.

Furthermore, cultural transformation because of the globalization can also be another major factor for the decline of such indigenous practices. It is also mentioned that there is no formal way to transfer this form of knowledge from one generation to the next. As to the elders saying, children do not like their own custom or tradition more inclining to the modern. It is also evident that less emphasis given to the practice by concerned body by itself is another responsible factor for the decline of such environmental friendly practice. This is found agreeable with [38] finding that states acceleration of globalization has really permeated African customs, norms and values and changed the cultural thinking of Africans towards western perspectives [39]. Similarly confirmed that the globalization changed not only the culture of indigenous people but also impacted continued future of the societies themselves [40,41]. Study found no difference with respect to the impact of globalization on the norms and beliefs of the indigenous community [42]. has also bolds stated that the negative effects of globalization have been much more than its positive effects on Ethiopian cultures. Therefore, it happen to be clear that indigenous practices has been subjected to the influence of globalization, and hence, no exception applies to indigenous tree conservation practices.

## 4 Conclusion and recommendation

Indigenous trees conservation of Sidama which contributed to the sustainability of the environment for centuries has been facing the fate of deterioration currently. The major challenges for the sustainability of the practice were found to be massive conversion of religion from traditional to the Christianity predominantly to Protestantism. Decline in economic production of land accentuated by fast growing population has its own footprint in the on the decline of the deep rooted knowledge of the community. Ignorance by the experts regarding societal practices in formulating of policies, strategies and most education road map and curricula have exerted the challenge to the continuity of the indigenous practices. Owing to the fact of declining environmental friendly practices, the state and quality of the environment is shrinking which is manifested by increasing loss of soil fertility, high variability of micro climate in the vicinity mainly declining rainfall and loss of vegetation diversity of species. This in turn, is expected to influence the production capacity of the farmers deepening their low level of living eventually affecting the overall production and productivity of the country at large.

Based on the above conclusion, the following actions are forward as recommendation for consideration.

➢ Indigenous knowledge have a great contribution for the management of the environment and therefore, the government of Ethiopia should give the room it in during enactment of rule and policies as well as it implementation;
➢ Great attention should be given to the creation of the awareness about the significance of the indigenous practices to social as well as ecological values;
➢ In depth discussion has to be addressed by scholars, administrative bodies and religious leaders;
➢ Education system should equally treat local based knowledge so as integrated the scientific understanding with the existing practices of local community.

## Author contribution statement

Tirfu Kakiso Kakawa: Conceived and designed the experiments; Performed the experiments; Analyzed and interpreted the data; Contributed reagents, materials, analysis tools or data; Wrote the paper.

## Data availability statement

Data will be made available on request.

## Declaration of interest's statement

The authors declare no conflict of interest.
